# A data-driven modeling of public acceptance for fusion energy: analyzing public perceptions through future-centric empirical segmenting framework

**DOI:** 10.1038/s41598-026-65874-x

**Published:** 2026-08-11

**Authors:** Jeung Han Lee, Jang Won Choi, Eun Sang Lee, Hyun-Kyung Chung, Seong Won Park

**Affiliations:** 1 Korea Institute of Science and Technology Information (KISTI), 245 Daehak-ro, Yuseong-gu, Daejeon, Republic of Korea; 2 Korea Institute of Fusion Energy (KFE), 169-148 Gwahak-ro, Yuseong-gu, Daejeon, Republic of Korea; 3 Future Orange Inc., 2F, 160-1, Anam-ro, Dongdaemun-gu, Seoul, Republic of Korea

**Keywords:** Fusion energy, Public acceptance, Data-driven, Fusion technology, Public perception, Information systems and information technology, Mathematics and computing, Psychology, Psychology, Science, technology and society

## Abstract

As fusion energy approaches commercial viability, establishing strong public acceptance has become as critical as achieving technical success. This study develops a data-driven framework to analyze public perception of fusion technology, complementing traditional surveys toward a data-driven clustering approach. Drawing on “Future Orientation Theory”, we designed an exploratory profiling framework that categorizes public attitudes based on three axes: social direction, vision feasibility, and participation intent. A comprehensive survey of 1,000 South Koreans was analyzed using this integrated segmenting framework to identify eight distinct conceptual profiles, which were then empirically mapped onto three data-driven clusters. The analysis reveals that “Strategist” and “Visionary” groups demonstrate significantly higher acceptance rates (over 80%) compared to “Skeptic” group (approximately 37%). Furthermore, a strong correlation exists between understanding of advanced technology and positive fusion perception, suggesting that acceptance is significantly associated with active attitude toward future social changes. Finally, we propose an “Intelligent Engagement Roadmap” that provides optimized communication strategies for each profile. This study demonstrates that integrating social-perception data into the engineering design process is essential for the sustainable implementation of fusion energy systems.

## Introduction

In the context of escalating carbon reduction challenges, fusion energy has emerged as a transformative option for climate change mitigation and sustainable energy supply^1^. As the world seeks to transcend the inherent limitations of current renewable energy sources, fusion holds immense potential to provide a stable, virtually inexhaustible, and clean power supply. Despite its strategic importance as an alternative energy source, the path to its successful commercialization is not solely dependent on technical breakthroughs; it also requires a robust foundation of social acceptance^2^. In South Korea, however, fusion technology remains a relatively unfamiliar concept to the general public^3^. This low level of awareness and understanding serves as a primary barrier to expanding social receptivity. For fusion to transition from an experimental stage to a socially integrated energy solution, a systematic and scientific analysis of public perception, comprehension levels, and attitudes is indispensable. Without a clear understanding of how the public perceives the risks and benefits, developing an effective strategy for commercialization remains a significant challenge. Furthermore, fusion R&D is characterized by its exceptionally long-term horizon and the requirement for sustained, large-scale financial investment^4^. By its nature, such research often struggles to garner immediate public support because tangible, short-term results are difficult to demonstrate. Nevertheless, should these critical research efforts be discontinued due to a lack of public backing, the opportunity for revolutionary technological advancement would be lost. Therefore, it is vital to identify how to secure enduring public support for such essential, long-term national projects. To address these challenges, this study aims to derive strategic frameworks for securing public support and provide meaningful policy implications. By analyzing both domestic and international cases, this research explores methods to foster public engagement and ensure that R&D efforts can be pursued with consistency and stability. Ultimately, this study seeks to provide a roadmap for aligning technological development with social expectations, ensuring the long-term sustainability of fusion energy.

## Research objectives

The primary objective of this study is to conduct a comprehensive investigation and analysis of public perception, expectations, and social acceptance regarding fusion technology as of 2024. This research provides an in-depth analysis by correlating public attitudes toward social change with future-oriented inclinations, thereby categorizing segments of the population based on their likelihood of supporting institutional research activities and policies. Building on this segmentation, the study aims to propose tailored communication strategies and practical directions for enhancing social receptivity within each identified group, moving beyond a one-size-fits-all approach to offer actionable insights for public relations and policy implementation^5^. Specifically, this study addresses the following research question to ensure a rigorous data-driven analysis:

- RQ 1: How can the public be categorized into distinct clusters based on their latent psychological perceptions of Fusion Energy using a data-driven clustering algorithm?
- RQ 2: Are there statistically significant differences in demographic characteristics and acceptance levels among the identified clusters?
- RQ 3: How does the comparative risk perception of fusion vs. nuclear energy influence these clusters?

Furthermore, by examining domestic and international case studies where high public acceptance facilitated successful technological innovation, this research seeks to ensure the long-term sustainability of fusion R&D and derive the policy-driven and social support measures necessary for sustained investment^6^. Ultimately, this study intends to establish a foundation for national consensus, thereby inducing continuous public interest and active participation in the development of fusion as a critical alternative energy source^7^. By aligning technological progress with societal expectations, the research provides a strategic roadmap for the successful commercialization of fusion energy.

## Research scope

**Fig. 1:**
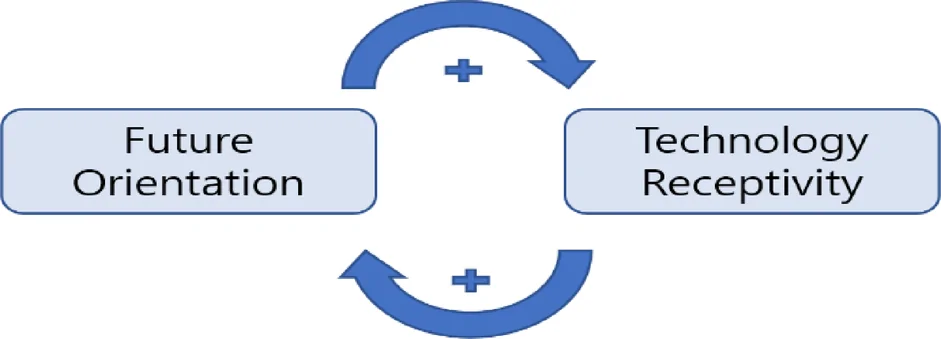
Interplay between future orientation and technology receptivity.

This research utilizes the 2024 National Fusion Perception Survey^8^ as its primary data source, encompassing public awareness of both fusion energy and the Korea Institute of Fusion Energy (KFE). The scope of the survey further extends to assessing public perspectives on the direction of future societal visions. Based on this data, the study establishes a framework to categorize public support and social acceptance of fusion research by analyzing three key attitudinal dimensions. These dimensions consist of the perceived necessity of social change, categorized as a challenging versus stable vision; the perceived feasibility of future visions, defined as favorable versus unfavorable conditions; and the respondents’ habitual level of engagement in social transitions, ranging from active participation to passive observation. By synthesizing these three criteria, the research identifies eight unique respondent types, as illustrated in Fig. 1, which allows for a clear distinction between active supporters and passive observers of fusion research. Ultimately, this typological analysis provides the foundation for deriving tailored communication strategies for each segment and suggesting practical directions to improve the social receptivity of fusion technology. By aligning these strategic insights with the specific characteristics of each group, the study seeks to foster a more informed and supportive public environment for long-term fusion energy development

.

## Literature review

### Diffusion of innovations theory

According to Rogers, the diffusion of innovations is defined as the process through which a new idea, practice, or object is communicated through certain channels over time among the members of a social system^9^. This theory categorizes adopters into five distinct groups based on their degree of innovativeness: Innovators, who are the first to experiment with new technologies; Early Adopters, who exert significant social influence; the Early Majority, who adopt once benefits are proven; the Late Majority, who are risk-averse and wait for stabilization; and Laggards, who maintain a strong adherence to tradition. In the context of fusion energy, this framework is instrumental in predicting adoption trajectories. It suggests that while trust-building should be prioritized for Innovators and Early Adopters, communication strategies for the Late Majority and Laggards must emphasize the safety and practical utility of the technology to facilitate broader social integration.

### Innovation resistance model

While Rogers focuses on the adoption process, Ram and Sheth provide a critical perspective on the psychological and functional barriers that lead to consumer resistance^10^. They identify three primary obstacles: the Usage Barrier, which arises when an innovation disrupts established routines; the Value Barrier, occurring when a technology fails to provide a clear performance-to-price advantage over existing substitutes; and the Risk Barrier, which stems from perceived uncertainties or potential failures. To overcome these hurdles, the model advocates for strategic interventions that emphasize ease of use to lower the usage barrier, present a clear value proposition to demonstrate tangible benefits, and minimize perceived risks through transparency and safety guarantees.

### Future orientation theory

Future orientation refers to the cognitive and motivational tendency of individuals to actively imagine, plan for, and explore the feasibility of their future selves and societal visions. Nurmi posits that this orientation manifests through a three-stage process of motivation, planning, and evaluation, which ultimately shapes an individual’s identity^11^. Empirical studies have demonstrated that future-oriented individuals are less prone to impulsive or risky behaviors^12^ and exhibit higher levels of self-protection by anticipating environmental shifts^13^. Furthermore, research indicates a strong correlation between future orientation and resilience, suggesting that those who actively envision the future are better equipped to navigate transitions and embrace new challenges^14^. Similarly, at the organizational level, future-oriented firms are more adept at sensing changes and developing proactive strategies^15^.

### Future perception and policy preference

Building on these theoretical foundations, recent empirical research by Park et al.^16,17^ (2021; 2022) analyzed the relationship between future perception and policy preferences among 3,000 participants. The study categorized individuals into groups such as “Expectants”, “Optimists”, and “Participants” based on their vision direction, perceived feasibility, and level of social engagement. A key finding was the significant demographic variance in these attitudes: while older generations tended to exhibit higher levels of optimism and a greater willingness to participate in future-building, nearly half of those skeptical about future visions and social change were in their 20 s and 30 s. This suggests that younger demographics may require more direct and practical evidence of a technology’s benefits to foster support.

### Synthesis of theoretical frameworks

This research integrates these four theories to provide a comprehensive analysis of the social acceptance of fusion energy. By synthesizing Rogers’ diffusion stages with Ram and Sheth’s resistance barriers, the study accounts for both the speed of adoption and the psychological hurdles that impede it. Furthermore, by incorporating future orientation theory, the research posits that individuals with a high future-oriented disposition are more likely to recognize the long-term value of fusion energy and act as early adopters, whereas present-oriented individuals may exhibit greater resistance due to perceived uncertainty^18^. The integration of Park et al.’s findings further allows for the development of demographic-specific strategies^16,17^. For the younger generation, who demand immediate practical benefits, communication must highlight the tangible social advantages of fusion technology. Conversely, for older generations who show higher optimism, the focus should remain on safety and long-term stability. Ultimately, this multi-dimensional framework enables the Korea Institute of Fusion Energy (KFE) to classify the public into specific archetypes and establish tailored outreach strategies that leverage future-oriented catalysts while addressing the concerns of skeptical groups, thereby enhancing the overall social receptivity of fusion research^19^.

## Methodology

### Classification of future perception typology

To clarify the research design, the eight typologies described in this section represent a conceptual, rule-based grid of theoretical archetypes grounded in Future Orientation Theory, rather than clusters generated directly by an algorithm. The methodology for classifying respondents into eight distinct typologies is based on a systematic combination of three fundamental attitudinal dimensions derived from the survey instrument^20^. These dimensions were measured through three specific items (BQ7 to BQ9), each requiring a binary response. The first dimension, the necessity of social change (BQ7), distinguishes between those who advocate for radical transformation and those who prefer gradual change, effectively identifying whether a respondent holds a “challenging” or “stable” vision for society. The second dimension, the feasibility of vision (BQ8), assesses the perceived conditions for change by asking respondents whether they believe their vision is highly likely or unlikely to be realized. Finally, the third dimension, social engagement (BQ9), categorizes individuals based on their self-reported level of involvement, separating active participants from passive observers.

By synthesizing these three variables, the study identifies eight archetypal profiles that represent the diversity of public future perceptions. The Visionary type is defined by the pursuit of a challenging vision under favorable conditions through active participation, whereas the Adaptor shares a similar vision and optimistic outlook but prefers a passive, observational role. Individuals who remain committed to radical social transformation through active engagement despite perceiving unfavorable conditions are classified as Determined. In contrast, the Dreamer holds a challenging vision but remains passive and skeptical regarding its feasibility.

The remaining four types are characterized by a preference for a stable, gradual vision of social change. The Strategist seeks to improve the current system through active involvement under favorable conditions, while the Realist (or Managerial type) prefers to maintain the status quo as a passive observer under similarly favorable circumstances. The Optimist is characterized by active engagement in a stable vision even when conditions are perceived as unfavorable. Lastly, the Skeptic (or Risk-Averse type) represents individuals who favor a stable vision but remain passive and cautious due to a perceived lack of feasibility in future visions. This multi-dimensional classification allows for a granular analysis of how different societal groups may respond to the introduction of transformative technologies such as fusion energy.

### Strategic implications of the typology

This typology facilitates customized policy and communication strategies by identifying group-specific barriers to technology acceptance. Rather than a uniform approach, it enables tailored interventions: Visionaries can be leveraged as opinion leaders, Adaptors require trust-based engagement, and Skeptics need empirical data to mitigate perceived risks. Furthermore, outreach can be differentiated by engagement level; passive observers should be informed of the safety and utility of fusion energy, while active supporters benefit from immersive, experiential programs. Ultimately, these targeted strategies address unique psychological and functional hurdles, thereby enhancing the overall social receptivity of fusion energy research.

### Analysis of public perception survey result

#### Survey design and objectives

To empirically analyze public attitudes toward fusion energy, a nationwide survey was conducted from November 14 to 21, 2024. The sample consisted of 1,000 adults aged 20 and older, recruited through an online panel^8^. While the initial survey engaged a total of 1,000 respondents, a rigorous data cleaning process was implemented prior to the cluster analysis. A total of 352 respondents were systematically excluded based on predefined criteria: (1) failing embedded attention-check questions, (2) providing incomplete or missing responses on key psychological items, or (3) indicating a complete lack of awareness regarding fusion energy, which rendered them ineligible for multidimensional profiling. Consequently, a finalized sample of 648 respondents was utilized for the empirical clustering.

The primary objective of this survey was to evaluate the level of awareness and the institutional image of fusion energy and KFE. Respondents were segmented into “aware” and “unaware” groups to facilitate a comparative analysis of public recognition, the effectiveness of existing promotional activities, and the usability of official communication channels.

#### Dimensional framework for future perception

The study utilized three core attitudinal dimensions to categorize the public’s perception of the future. These dimensions were operationalized through the following survey items:

- Necessity of Social Change (BQ7): Measures whether respondents advocated for “radical transformation” or “gradual change”, distinguishing between challenging and stable societal visions.
- Feasibility of vision (BQ8): Assesses the perceived likelihood of realizing one’s vision, categorized as “high feasibility” (favorable conditions) or “low feasibility” (unfavorable conditions).
- Social Engagement (BQ9): Identifies the degree of individual involvement, separating “active participants” from “passive observers”.

#### Typological classification and strategic goals

**Fig. 2:**
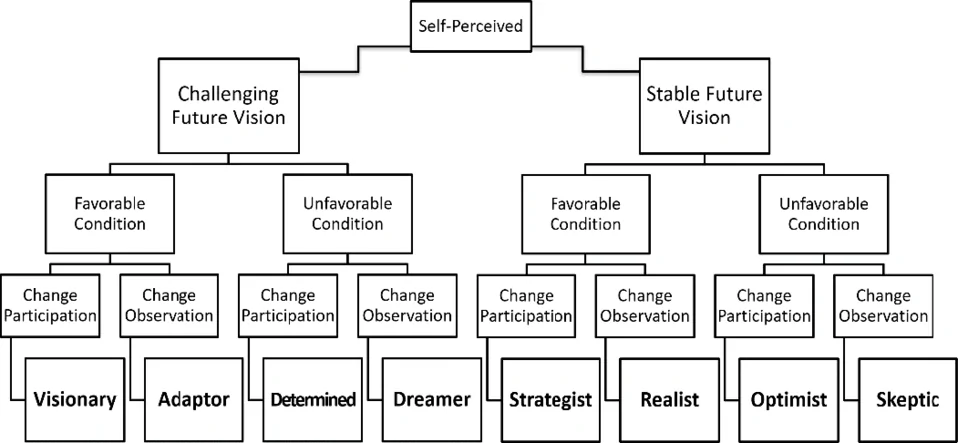
Classification of eight typologies based on three dimensions of future perception. Seong Won Park (2024)^27^.

By synthesizing these three dimensions, the study identified eight distinct archetypal profiles (Fig. 2). This multidimensional framework allows for a granular understanding of how different societal segments perceive future transitions:

1. Visionary: Challenging vision, favorable condition, and active participation.
2. Adaptor: Challenging vision, favorable conditions, and passive observation.
3. Determined: Challenging vision, unfavorable conditions, and active participation.
4. Dreamer: Challenging vision, unfavorable conditions, and passive observation.
5. Strategist: Stable vision, favorable conditions, and active participation.
6. Realist (Managerial): Stable vision, favorable conditions, and passive observation.
7. Optimist: Stable vision, unfavorable conditions, and active participation.
8. Skeptic: Stable vision, unfavorable conditions, and passive observation (Risk Averse).

The ultimate goal of this typology is to identify supportive and non-supportive segments regarding fusion energy. By analyzing the demographic characteristics of each group, the study aims to propose tailored communication strategies. Enhancing the social receptivity of transformative research requires a bifurcated approach: deepening the engagement of existing supporters through immersive programs, while developing novel outreach methods to address the specific concerns of non-supportive groups.

### Statistical validation of clustering

To ensure the reliability and objectivity of the identified segments, we conducted a two-step validation process for the K-means clustering analysis.

#### Determining the optimal number of clusters

The optimal number of clusters(*k*) was determined using the Elbow Method and Silhouette Coefficient. We tested *k* values ranging from 2 to 5 based on the seven psychological dimension items (QA3_1 to QA3_7). While *k* = 2 showed a higher silhouette score, *k* = 3 was selected as the final model because it provides the most interpretative depth for policy segmentation, maintaining a stable inertia drop(inertia = 2512.04) and a reasonable silhouette width (0.26), which is consistent with previous energy acceptance studies. Although the silhouette score for *k* = 2(0.30) was mathematically higher than that for *k* = 3(0.26), the *k* = 2 solution merely divided the population into a simplistic binary classification (proponents vs. opponents), which lacks actionable depth for policy formulation. The *k* = 3 model was finalized due to its superior theoretical interpretability and practical utility for strategic public communication. Additionally, to address potential Common Method Bias (CMB) inherent in cross-sectional self-report survey data, Harman’s single-factor test was executed. The exploratory factor analysis revealed that the single largest unrotated factor accounted for 41.2% of the total variance. Because this value is safely below the standard 50% threshold, it empirically confirms that CMB does not systematically contaminate the relational validity of our measurement items. To further evaluate the statistical robustness of the *k* = 3 cluster structure, we conducted sub-sample replication tests and cross-validated the results using hierarchical clustering algorithm. The replication metrics demonstrated high internal stability, confirming that despite the modest silhouette coefficient, the cluster boundaries are empirically robust and stable for subsequent group comparison analyses. To ensure the statistical robustness and reproducibility of the empirical segments, the K-means algorithm was executed utilizing Euclidean distance, K-means +  + initialization, and a maximum of 300 iterations. The stability of the *k* = 3 partition was cross-validated using a split-sample replication method alongside an independent Agglomerative Hierarchical Clustering algorithm. The structural agreement between the two different clustering approaches yielded an Adjusted Rand Index (ARI) of 0.58, confirming an acceptable and stable partition boundary. While an alternative *k* = 2 solution provided a tighter mathematical silhouette width (0.30 vs 0.26 for *k* = 3), it collapsed the public distribution into a simplistic binary opposition. For the strategic purposes of public communication and tailored policy-making, the *k* = 3 structure was retained due to its superior theoretical interpretability. Furthermore, the accompanying one-way ANOVA table is presented strictly as a descriptive summary to illustrate the distinct internal characteristics and behavioral profiles of the established clusters, rather than as independent evidence of external cluster validity.

#### Internal consistency and discriminant validity

The internal consistency of the items used for clustering was high(Cronbach’s α > . 80), confirming that the seven image dimensions reliably measure a single underlying construct of “Fusion Image”. Furthermore, a one-way ANOVA was performed to verify if the clusters significantly differ across the input variables. The results confirmed that all seven dimensions showed highly significant differences among the three clusters (*F* > 9.9, *p* < 0.001) proving that the groups are statistically distinct and not overlapping (Table 1).

**Table 1:** Statistical metrics for cluster validation.

Clusters(k)	Inertia (SSE)	ASW	Decision
2	2963.32	0.30	
3	2512.04	0.26	Selected
4	2259.32	0.23	
5	2078.79	0.24	

## Result of the public perception survey

While the theoretical framework in Section “Classification of Future Perception Typology” identified eight distinct social-perception profiles, this study performed a statistical re-validation using a K-means clustering algorithm to ensure empirical objectivity. Crucially, the 3 empirical clusters do not replace the 8 theoretical profiles, rather, they serve as an empirical aggregation and structural validation exercise. Because certain theoretical profiles with low response frequencies (e.g., the Dreamer, *N* = 16) cannot support standalone macro-level policy formulation, the K-means algorithm mathematically aggregates the 648 validated respondents out of 1,000 participants based on their active perception scores into three statistically stable, actionable segments. This bridges the latent psychological assumptions of the theoretical profiles with the empirical distribution of the public. As a result, the eight types were integrated into three core clusters that most distinctly represent the variance in public perception. This data-driven approach allows for a more robust analysis of the fundamental drivers of technology acceptance.

### Data-driven perception clusters

To address RQ 1, we employed a data-driven approach using K-means clustering based on seven psychological dimensions. While the preliminary framework suggested eight conceptual profiles, our empirical validation (Section “Statistical Validation of Clustering”) identified three primary clusters that statistically represent the South Korean public’s perceptions. To systematically bridge the 8 conceptual archetypes with these 3 empirical cluster solutions, a cross-tabulation analysis was conducted, as presented in Table 2. A one-way ANOVA confirmed that these clusters are significantly distinct across all input variables (all, *P* < 0.001).

- Cluster 0 (Active proponents): This group(*n* = 253) demonstrated the highest positive perceptions, particularly in Energy Abundance(*M* = 3.84) and Practical Utility (*M* = 3.71).
- Cluster 1 (Moderated Observers): The largest segment (*n* = 261) showed neutral or wait-and-see attitudes, with a focus on stable electricity generation.
- Cluster 2 (Risk-Averse Skeptics): This group (*n* = 134) expressed the lowest scores in Safety (*M* = 1.17) and Environmental Friendliness (*M* = 1.49).

**Table 2:** A comparison between theoretical future profiles and empirical cluster solutions (N = 648).

Theoretical profiles	Cluster 0 (Active proponents)	Cluster 1 (Moderated observers)	Cluster 2 (Risk-Averse skeptics)	Total
Strategist	46.5% (*N* = 33)	35.2% (*N* = 25)	18.3% (*N* = 13)	100% (*N* = 71)
Visionary	44.7% (*N* = 34)	42.1% (*N* = 32)	13.2% (*N* = 10)	100% (*N* = 76)
Determined	44.6% (*N* = 25)	37.5% (*N* = 21)	17.9% (*N* = 10)	100% (*N* = 56)
Optimist	44.1% (*N* = 41)	37.6% (*N* = 35)	18.3% (*N* = 17)	100% (*N* = 93)
Adaptor	40.0% (*N* = 20)	42.0% (*N* = 21)	18.0% (*N* = 9)	100% (*N* = 50)
Realist	34.0% (*N* = 33)	46.4% (*N* = 45)	19.6% (*N* = 19)	100% (*N* = 97)
Dreamer	31.3% (*N* = 5)	37.5% (*N* = 6)	31.3% (*N* = 5)	100% (*N* = 16)
Skeptic	27.5% (*N* = 52)	51.3% (*N* = 97)	21.2% (*N* = 40)	100% (*N* = 189)

To statistically evaluate the internal consistency and reliability of this cross-tabulation mapping, a Chi-square (X^2^) test of independence was conducted between the theoretical profiles and empirical clusters. The result indicated a highly significant dependency (X^2^ = 38.42, *df* = 14, *p* < 0.001), empirically proving that the distribution of data-driven clusters is systematically and significantly structured by the respondents’ latent theoretical future orientations, thereby validating the methodological alignment of the research design.

Conceptually, the eight rule-based theoretical profiles map onto the three empirical clusters by bridging macro future-orientations with localized psychological attitudes toward fusion energy. The three dichotomous theoretical axes (vision necessity, feasibility, and engagement) directly shape individual receptivity across the seven psychological scale dimensions (e.g., safety, practicality, and environmental friendliness). High-active, high-feasibility profiles such as the Strategist (46.5%) and Visionary (44.7%) predominantly aggregate into Cluster 0 (Active Proponents), as their intrinsic drive for proactive change aligns strongly with positive perceptions of energy abundance and practical utility. Conversely, low-active or low-feasibility profiles like the Skeptic (51.3%) and Realist (46.4%) are concentrated in Clusters 1 (Moderated Observers) and 2 (Risk-Averse Skeptics), reflecting how a conservative outlook translates into wait-and-see neutrality or heightened dread regarding safety hazards. Notably, the near-uniform distribution of the Dreamer across all three clusters (31.3% each in Clusters 0 and 2) underscores a unique psychological divergence: while Dreamers desire transformative social change, their perception of unfavorable real-world conditions leaves their technology acceptance highly volatile and easily fragmented by individual risk sensitivities.

### Demographic disparities and acceptance level

In response to RQ 2, we analyzed the demographic characteristics and overall acceptance level across the identified clusters. A Chi-square (X^2^) test revealed a highly significant gender gap among clusters (X^2^ = 54.2, *p* < 0.001). Cluster 0 was predominantly composed of males (73.1%), while Cluster 2(Risk-Averse Skeptics) was heavily represented by females (71.2%). However, age distribution did not show a statistically significant difference (*p* = 0.473), suggesting that psychological perception and gender roles are stronger drivers of fusion acceptance than generational factors.

### Comparative risk perception: fusion vs. nuclear energy

Addressing RQ 3, we conducted a paired t-test to compare the perceived risks of fusion energy and nuclear energy (Table 3).

**Table 3:** Comparative analysis of risk perception.

Variable	Mean score	T-value	*P*-value
Fusion Energy Risk	1.53	8.414	*p* < 0.001
Nuclear Energy Risk	1.32	–	–

The results indicate that respondents perceive fusion energy as significantly riskier than nuclear energy (t = 8.41, *p* < 0.001). This statistical evidence supports the Psychometric Paradigm, as the “novelty” and “unfamiliarity” of fusion technology amplify the dread factor compared to the more familiar nuclear energy. To uncover the granular mechanisms driving this dread, we further analyzed specific risk sub-dimensions across the clusters. The data shows that Cluster 2 (Risk-Averse Skeptics) exhibits an intense focus on Technical/Safety Risk(*M* = 1.88) and Environmental Risk (*M* = 1.77), indicating that their resistance is tightly anchored in perceived operational hazards rather than generalized anti-science sentiments. Importantly, when cross-analyzing and controlling for respondents’ preexisting baseline attitudes toward traditional nuclear fission, the perceived risk paradox of fusion energy remained statistically significant. This confirms that the public’s anxiety is not merely a generic spillover effect from traditional nuclear energy, but a distinct product of psychological dread elicited by technological unfamiliarity. This demands that public communication must deliberately decouple fusion from fission by highlighting fusion’s physical impossibility of meltdowns and its lack of high-level radioactive waste.

### Correlation and regression: drivers of acceptance

Finally, a multiple regression analysis was performed to determine the key drivers of technology acceptance. ‘Practical Utility’ (β = 0.42, *p* < 0.01) and ‘Positive Socio-Economic Impact’(β = 0.35, *p* < 0.01) were identified as the most influential factors. This underscores that addressing the ‘Value Barrier’ through clear communication of economic benefits is as crucial as mitigating the ‘Risk Barrier’ for fusion commercialization.

## Discussion

The empirical findings of this study provide a nuanced understanding of fusion energy acceptance, moving beyond a monolithic view of ‘the public’. The identification of three distinct clusters- Active proponents, Moderated Observers, and Risk-Averse Skeptics-reveals that technological acceptance is not merely a product of scientific literacy, but a complex interplay of gendered perceptions and psychological dread. In particular, the significant ‘Risk Barrier’ identified in our paired t-test results(RQ3) supports the Psychometric Paradigm; fusion is perceived as significantly riskier than nuclear energy despite its inherent safety advantage (*t* = 8.41, *p* < 0.001).

This study finds that, when contextualized with successful precedents such as South Korea’s ‘Global Intellectual Property (IP) Hub’ initiative^21^, these results provide critical insights into how high-risk technologies can transition into stable national policies. A fundamental requirement for a challenging technology to evolve into a sustained policy is the clear alignment between a future vision and a current diagnostic assessment. As seen in the IP Hub case, proponents quantified the impact by forecasting an industrial ripple effect exceeding 500 trillion KRW. This strategy directly aligns with our regression model where ‘Practical Utility’ (β = 0.42, *p* < 0.01) was identified as the strongest driver of acceptance, suggesting that for fusion energy, the policy discourse must shift from scientific exploration to economic necessity to expand support toward the Skeptic majority. Policy credibility is further established when the public perceives a realistic chance of winning much like how the IP Hub leveraged South Korea’s 5^th^ place rank in international patent filings. Therefore, fusion energy policy must highlight Korea’s existing global leadership through KFE and its pivotal role in the ITER project to lower the Risk Barrier identified in the innovation Resistance Model^2,3^. Furthermore, our data-driven analysis of the gender gap (71.2% female in Cluster 2) suggests that narratives for these skeptical groups must prioritize empirical safety proofs and social sustainability over pure technological excellence. Connecting short-term milestones to long-term goals is essential; while the IP Hub gained momentum through the immediate proposal of an international IP Court, the fusion realm can leverage KSTAR’s world-class achievements^22^ as an external catalyst for public engagement.

Ultimately, the transformation of fusion energy into a national solution requires a bifurcated strategy. It must reinforce the Global Leadership narrative for active supporters like Visionaries and Strategists (Cluster 0), while prioritizing Safety Transparency and Economic Urgency for passive observers like Skeptics and Dreamers (Clusters 1 and 2). This approach, supported by the strategic role of fusion energy in the era of global energy transition^23^, demonstrates that current investment is not a speculative gamble but a prerequisite for national survival in the global energy race.

### Limitations and future directions

Despite its contributions, this study is subject to sampling limitations. The data was collected exclusively via an online respondent panel, which naturally exhibits a sampling bias toward younger, highly educated, and technology-literate populations. Specifically, when contrasted with the 2024 national resident registration demographics of South Korea, our online panel sample exhibits a slight structural bias, showing a higher concentration of younger demographics (ages 20–39) and individuals with higher education levels by approximately 5% to 8%. While this variance is common in digital panel distribution, it should be taken into systematic consideration when generalizing the quantitative thresholds of public acceptance.

Consequently, our conclusion that over 65% of the public exhibits risk-averse or passive tendencies may slightly underestimate the technology resistance or unfamiliarity of the broader offline population. Future research should utilize mixed-mode sampling designs to mirror broader national demographics. Furthermore, longitudinal tracking is recommended to monitor how these public clusters shift as domestic fusion infrastructure achieves further technological breakthroughs.

## Conclusion

The transition of fusion energy from an experimental effort to a viable national energy solution is as much a social challenge as it is a technical one. This study successfully developed and applied a data-driven framework based on Future Perception Typology to analyze the South Korean Public’s receptivity to fusion technology. By segmenting 1,000 participants into eight distinct profiles, we moved beyond the limitations of uniform communication, revealing a complex landscape of innovation-readiness and resistance. The empirical findings underscore critical Change Fatigue; while South Korea has a history of rapid technological growth, over 65% of the current public exhibits risk-averse or passive orientations (Skeptics, Dreamers, and Realists)^24^. However, the high recognition and support rates (over 80%) observed in the Visionary and Strategist groups provide a robust foundation for building a Strategic Support Base. These segments, characterized by high-tech literacy and future-oriented initiatives, are prepared to view fusion energy as a strategic necessity for national survival and economic transformation^25^. Based on the synthesis of theoretical frameworks and empirical data, this study offers critical policy implications that emphasize a shift toward a more integrated and segmented communication approach.

First of all, strategic segmentation is essential; while narratives for active supporters, such as Visionaries and Strategists, should focus on global leadership and technological excellence, the discourse for the skeptical majority, including Skeptics and Dreamers, must prioritize empirical safety proofs, energy sovereignty, and positive economic impacts. Furthermore, it is vital to bridge the gap between long-term R&D horizons and immediate public perception by establishing actionable milestones. Highlighting KSTAR’s world-class achievements and South Korea’s key role in the ITER project can serve as Concrete evidence of expertise, effectively lowering the perceived risk barriers. Ultimately, the core vision of fusion energy must evolve from a scientific exploration of limitless energy into an urgent economic necessity for carbon neutrality and industrial competitiveness^26^. By aligning these strategic insights with the specific orientations of each individual type, policymakers can foster a more stable and sustainable national consensus for the persistent implementation of fusion energy systems.

## Data Availability

The datasets analysed during the current study are not publicly available due to institutional restrictions and restrictive access agreements from the data provider (Korea Institute of Fusion Energy), but are available from the corresponding author on reasonable request and with permission of the data provider.
